# A new mathematical model for determining optimal workforce planning of pilots in an airline company

**DOI:** 10.1007/s40747-021-00386-x

**Published:** 2021-05-21

**Authors:** İbrahim Zeki Akyurt, Yusuf Kuvvetli, Muhammet Deveci, Harish Garg, Mert Yuzsever

**Affiliations:** 1 Turkish Airlines Flight Academy, 09120 Efeler, Aydin Turkey; 2 Department of Industrial Engineering, University of Cukurova, 01330 Adana, Turkey; 3 Department of Industrial Enginnering, Turkish Naval Academy, National Defence University, 34940 Istanbul, Turkey; 4 School of Mathematics, Thapar Institute of Engineering and Technology, Deemed University, Patiala, Punjab 147004 India; 5 Turkish Airlines Inc, 34149 Yesilkoy, Istanbul Turkey

**Keywords:** Airline **w**orkforce planning, Mathematical modeling, Pilots, Aircraft types, Mixed integer programming

## Abstract

This study aims to model a workforce-planning problem of pilot roles which include captain and first officer in an airline company and to make an efficient plan having maximal utilization of minimum workforce requirements. To tackle this problem, a mixed integer programming based a new mathematical model is proposed. The model considers different conditions such as employing pilots with different skill types, resignations, retirements, holidays of pilots, transitions between different skills regarding needs of the demands during the planning horizon. The application of the proposed approach is investigated using a case study with real-world data from an airline company in Turkey. The results show that a company can use transitions instead of new employment and this is a more suitable medium-term production and human resource planning decision.

## Introduction

Airline companies face a number of challenges in their endeavour to ensure high efficiency in their operations [1]. Problems in airline operations are complex and cannot provide planners or researchers with a model and solve them within a reasonable amount of time. As the size of the airline increases, its problems become more complex [2]. For this reason, decision problems are solved using a hierarchical framework, where long-term problems are solved less frequently than short-term ones [3]. Some advanced solution methodologies combine some successive problems in a hierarchical order to ensure better integration of these subsequent decision-making steps. In this way, the output of one stage constitutes the input of the other stage.

The manpower planning, which considers pilot staffing and tanning, is one of the most important and challenging problems facing major airlines [4]. It involves the process of balancing the number of employees to be employed with the number of jobs demanded over the planning horizon [5]. If an airline fails to manage this effectively, it cannot sustain itself in the highly competitive air transport market [6].

In recent years, a considerable rise has been noted regarding the number of airline planning problems owing to the increase in the number of passengers availing air transport in conjunction with the rapid growth of such companies [7]. To meet this demand, airlines consider not only airplane constraints but also several other factors such as human resource planning. One of the most critical problems/constraints in terms of competition brought by this growth is the one concerning manpower planning. Due to the rapid growth of the airline industry, most airlines have an increasing need for qualified and experienced pilots. The problem regarding strategic manpower planning is primarily a question of the timing of transition training for pilots from one pilot group to another and that of the recruitment of new pilots, in order to minimize the inadequacy of pilots and training costs [8]. When conducting manpower planning, airlines should (1) correctly estimate personnel needs based on future business plan requirements, (2) identify staff to meet these needs, and (3) plan training [4]. Managing it according to other production factors is much more difficult and complex issue. It is possible to carry out a planned and systematic work to manage the pilot manpower well to ensure high efficiency in its operations. The problem of manpower planning, which is one of these management problems, refers to the determination of the number of pilots who should be kept under demand constraints during the planning horizon, to be employed or to be trained.

Since the pilot is a valuable resource, airlines companies want to keep it to a minimum level. The way to do this is to have minimum employment, which enables airline companies to cover all the flights they need with the least number of pilots. Despite the high salaries of the pilots, unfortunately Turkey’s pilot resource is scarce. Recently, the number of pilots has been decreasing. Turkish Airlines companies are growing fast but foreign pilots are returning to their countries due to the declining exchange rates. In Asian countries, especially in China, the salaries are high from them, so they go there.

This study primarily aims to minimize the vital number of pilot employments while planning, training of pilots between fleets and annual leaves. Workforce planning in human resource management is a critical and essential component of airline operation management. Determining the optimal number of pilots while executing the scheduled flight operations is affected by various parameters such as pilot requirements, employment, training, resignations, etc. The number of pilots is also limited; therefore, the requirement of new pilots considering, training, retirements, etc. is very essential for an airline. This study aims to identify and model the problem of pilot workforce planning according to the skill types of the pilots concerned. For this purpose, a mathematical programming approach and solution to the planning problem are sought.

The main contributions of this paper are the following:

- The description and modeling of the hierarchical workforce planning of pilots (with different pilot roles which are first officer and captain).
- The proposition of a new mixed integer programming solution for the underlying pilot workforce planning.
- Testing the proposed model for real-world data from an airline company in Turkey.
- The optimal planning approach by considering many real-world constraints.
- There is also an annual leave constraint. The pilots must to take their annual leave each month according to a certain quota. Our model solves the distribution of these annual permits according to the annual leave payments to keep the number of pilots to a minimum.
- In this model, resignations and retirement are a separate limitation, which can be considered a leave employment. The resignation and retirement ratio are obtained from the last 12-month data. This is the input of the model and the deviations here are not decisive. In fact, due to the high need for a pilot, the lack of resources affect the solution of such a problem. This problem is addressed to use the pilot in the optimal way and to minimize the need for more of them.

The rest of the paper is organized as follows: “Civil aviation of Turkey and airline company” section introduces civil aviation of Turkey. Literature review of workforce planning is given in “Literature review” section. The airline workforce planning problem and the proposed mathematical modeling are explained in “Mathematical model” section. “Experimental results” section presents the experimental results and analysis. Finally, conclusion and discussion are presented in “Results” section.

## Civil aviation of Turkey and airline company

Turkey has one of the world’s of the four fastest growing civil aviation sectors over the last ten years [9]. The market in the sector has not yet reached the satisfaction, and it is predicted that the growth of the sector will continue in the coming years. The civil aviation sector consists not only of passengers, but also cargo transportation, maintenance, ground handling and support services. Global competition conditions in the aviation sector have made airlines a network carrier that makes international passenger transport, rather than just an airline that carries passengers in local markets. For example, one of the Turkey's biggest airlines, Turkish Airlines (THY), which flies to 108 countries, more than any other airline, currently has 337 aircraft. Moreover, also 170 aircraft orders, flying to 121 countries, more than 301 destinations over 250 international and 304 airports in the world. Turkish Airlines plans to increase the number of aircraft to 500 by increasing the target number of fleets in 2023. It aims to carry 69 million passengers in 2017, up from 62.7 million last years [10, 11].

## Literature review

In recent years, various studies have been published to deal with workforce planning problems, which have different areas such as military planning [12], class scheduling for pilot training [13], health workforce planning [14–18], domestic freight forwarding [19], call center workforce planning [20], academic radiography workforce [21], rotating workforce schedules [22], workforce planning in electrical distribution utilities [23], and planning for check-in counters at airports [24].

There is also a variety of solution methods have proposed ranging from exact and meta-heuristic approaches. Dijkstra et al. [25] described a decision support system that has been investigated for the aircraft maintenance personnel planning using an integer program. Ingolfsson et al. [26] developed a stationary independent period-by-period integer program for workforce planning that presents a flexible and extensible method. Fowler et al. [27] presented two linear programming (LP)-based heuristics and a solution area partitioning approach to reduce the computation time for workforce planning with worker differences. A genetic algorithm was applied as an alternative method to obtain better solutions and to compare with the proposed heuristic results. Seçkiner et al. [28] developed an integer programming model for the hierarchical workforce problem under the compressed working week. The model is based on the integer programming formula developed by Billionnet. The main idea of this article is to use the compressed work week to reduce labor costs. This situation is also suitable for application at the same time. Huang et al. [29] applied a mathematical model for the workforce capacity planning problem. Stolletz [24] developed for fortnightly tour scheduling problem with flexible employee contracts using a binary LP formulation. The different tasks of the hierarchical workforce planning problem are characterized by time-dependent demand. A 2-week linear programming formulation with flexible working contracts was developed for the two-week tour scheduling problem. This binary programming model is solved by CPLEX for optimality for real-world demand scenarios with different workforce sizes. Corominas et al. [30] proposed a mixed integer linear program that is designed to solve for workforce planning, including production, human resources and cash management decisions. Othman et al. [31] addressed a labor force planning (WP) model that includes some human aspects such as skills, training, and employee personality and motivation in their research. A multi objective non-linear programming model has been developed to reduce hiring, job redundancy, training and overtime costs to a minimum and to minimize the number of the most productive workers. The aim is to determine the number of employees, the number of workers trained and the number of overtime hours for each type of worker. Besides, a proposed model-based decision support system (DSS) is introduced. Özgüven and Sungur [5] presented a study of a hierarchical workforce scheduling problem in which a more qualified worker could replace a less skilled worker, but vice versa. Five mathematical models are discussed in this context. Hewitt et al. [32] proposed integer programming for solving non-linear workforce planning which is based on learning. Jennings and Shah [33] investigated workforce planning and technology installation optimisation for utilities, which provides an example in Great Britain. Bastian et al. [34] examined the cyber workforce planning under uncertainity using stochastic programming and robust optimization for US Army. Fidanova et al. [35] presented a hybrid ant colony optimization algorithm to solve the workforce problem. Other studies based on mathematical model in different fields are Ebadizadeh and Lezgi [36], Farokhi [37], Davtalab and Ebadizadeh [38], Sanei and Hassasi [39] and Canıtez et al. [40].

The regarding workforce planning is very hard to solve with traditional mathematical model. Therefore, this study presents a new mixed integer programming solution for workforce planning of pilots in an airline. The model includes skills, referred as hierarchical level (for each role which are first officer and captain) and to the aircraft that each pilot is entitled to fly. The model also includes the possibility of re-training pilots yet hired, so that they can acquire new skills to adapt to the requirements of the company.

## Mathematical model

**Fig. 1:**
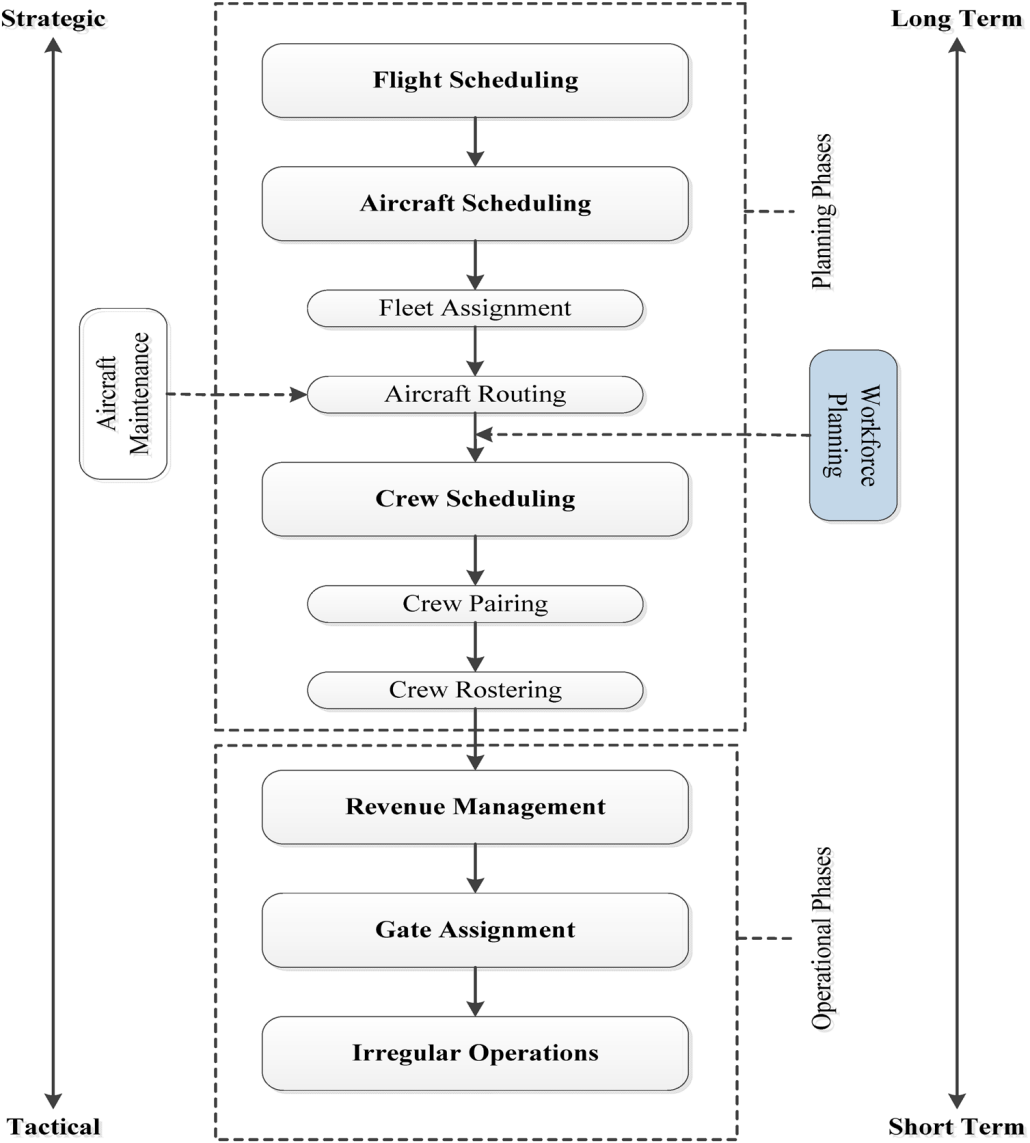
The hierarchy of airline planning

In this section, the description and information about the current operation of this problem, and the detailed mathematical formulation of the workforce planning problem are described. The hierarchy of airline planning [3] and workforce planning is also shown in Fig. 1.

### Problem description

In a typical airline company, a pilot can be hired as first officer or captain. Each aircraft type requires different skills, also requires first officer and captain recruitment. A first officer gets promoted to the role of captain by training and gaining experience. This makes the problem more interesting. The main aim of this problem is to find the optimal number of pilots for each role (first officer or captain) within every month during the planning horizon, which specifies the conditions such as demands, holidays, training, etc. Pilots have a skill, and they can flight regarding these skills. The planning horizon may start from the most demanded month. Due to the airline policy, holidays are divided into two parts which are summer and winter months.

When a new pilot is hired, the pilot needs some training before the flight. The number of graduates from the flight academy is determined by the airline policy. Some of the graduates may not be assigned a certain skill; therefore, it is required to determine these pilots skills.

Each pilot has a certain skill type; however, pilots may possess other skills during the planning horizon. The interchangeability between different skill types may be changed by the skill types.

**Fig. 2:**
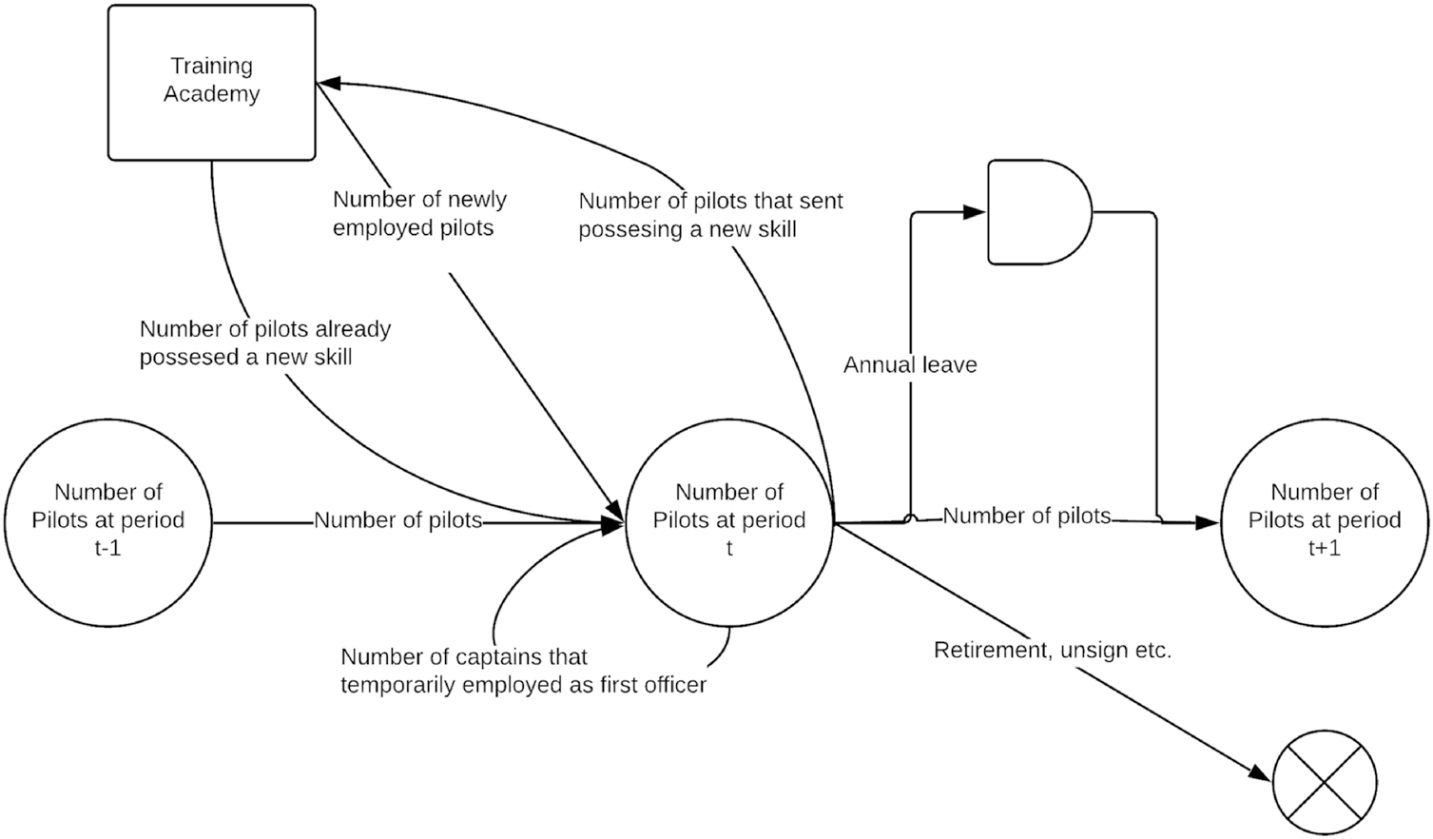
The statement of proposed model

The number of pilots may decrease permanently regarding retirements, resigns, changing skills. The pilot demands should be satisfied by providing a cost-effective employment plan; therefore, the company intends to employ less pilots according to these demands. The problem is stated in Fig. 2.

Pilots have basically two types of employment which are captains and first officers. Moreover, pilots have different skills regarding the aircraft types such as narrow-body and wide-body aircrafts. In case of requirement, captains can work as first officer role temporarily to find the best plan.

First of all, since the airline company does not have a workforce planning program, the transitions between types are made manually. There is one algorithm for annual leave and the constraint works according to quotas. The algorithm makes the distribution according to the constraint. This optimization program is not just based on the constraint, so that its staff does not fill up until the quota is full. In this minimum of employment, both annual leave and the number of manual types are reported to employment.

### Master model and notation

Now, the problem is formulated as a mixed-integer programming model. In this section, the master model and the necessary notations are given in Table 1.

**Table 1:** Notation for the mathematical model

Sets	Definition
*T*	Set of months which is indexed by *t*
*S*	Set of skills which is indexed by *s*
*F*, *C*	Set of first officer (*F*) and captain (*C*) skills,$F \cup C = S,F \cap C = \emptyset$
*W*, *V*	Set of winter (*W*) and summer (*V*)months,$W \cup V = T, W \cap V = \emptyset$

Parameter	Definition
*d*_*st*_	Pilot requirements for *s*th skill at *t*th month
*h*_*s*_	Number of available pilots for *s*th skill at starting month
$\alpha$	Training duration for a new employed pilot (months)
*A*_*st*_	Number of newly employed pilots with gaining *s*th skill at *t*th month
*R*_*st*_	Number of resigned, retired, etc. pilots for *s*th skill at *t*th month
$g_{{ss^{\prime } }}$	Training time requirements for transition from skill *s* to s′
$\beta_{s}^{1} ,\beta_{s}^{2} ,\beta_{s}^{3} ,\beta_{{ss^{\prime } }}^{4} ,\beta_{s}^{5}$	Penalty coefficients of number of pilots, new employments, temporary first officers, transition and annual leaves respectively
$\gamma$	Annual leaves balancing ratio
*Q*	Capacity of transition training academy
*K*_*t*_	Number of first officers currently trained and hired without skill at *t*th month
*L*_*t*_	Number of captains currently trained and hired without skill at *t*th month

Decision variable	Definition
*X*_*st*_	Number of pilots for *s*th skill at *t*th month
*Y*_*st*_	Number of pilots having *s*th skill taking annual leave at *t*th month
$Z_{{ss^{\prime } t}}$	Number of pilots having *s*th skill will be gained the *s*′th skill at *t*th month
*E*_*st*_	Number of pilots having *s*th skill newly employed and ready to flight at *t*th month
*B*_*st*_	Number of pilots having *s*th skill employed instead of first officer / captain at *t*th month
*M*_*st*_	Number of pilots having no skills and determined as *s*th skill at *t*th month

The assumptions of the proposed model are given follows:

- The airline company has a finite number of aircraft types which needs a finite number of skills.
- Pilots work as first officers or captains.
- There is a finite number of currently employed pilots at the start of the planning horizon. At the beginning of the planning horizon, pre-determined joins to the fleet, newly employed, currently employed in training without any skills, resigned and retired pilots are known in advance.
- The pilot requirements of each month regarding demand are known in advance.
- Training programs for new and transition pilots are known in advance. The training facility has a finite capacity.
- Annual leaves should be balanced during the planning horizon and summer and winter months conditions.

1 $$ \min z = \mathop \sum \limits_{t \in T} \mathop \sum \limits_{s \in S} \beta_{s}^{1} *X_{st} + \beta_{s}^{2} *E_{st} + \beta_{s}^{3} *B_{st} + \mathop \sum \limits_{{s^{\prime } \in S}} \beta_{{ss^{\prime}}}^{4} *Z_{{ss^{\prime } t}} + \beta_{s}^{5} *Y_{st} , $$

2 $$ \begin{aligned} & {\text{Subject to}} \\ & \begin{array}{*{20}l} {X_{st} - Y_{st} + B_{{s^{\prime}t}} \ge d_{st} } \hfill & {t \in T,s \in F,s^{\prime} \in C} \hfill \\ {} \hfill & {s^{\prime} = {\text{s}} + {1}} \hfill \\ \end{array} \\ \end{aligned} $$

3 $$ X_{st} - Y_{st} - B_{st} \ge d_{st} \quad t \in T,s \in C, $$

4 $$ \mathop \sum \limits_{t \in W} Y_{st} \ge h_{s} \times 0.5\quad s \in S, $$

5 $$ \mathop \sum \limits_{t \in V} Y_{st} \ge h_{s} \times 0.5\quad s \in S, $$

6 $$ Y_{st} \ge h_{s} \times \gamma ,t \in T,s \in S, $$

7 $$ X_{s1} = h_{s} + A_{s1} + M_{s1} - R_{s1} \quad s \in S, $$

8 $$ \begin{aligned} X_{st} & = X_{st - 1 } + A_{st} + E_{st - \propto } + M_{st} - \mathop \sum \limits_{{\begin{array}{*{20}c} {s^{\prime} \in S} \\ {} \\ {g_{{ss^{\prime}}} > 0} \\ \end{array} }} Z_{{ss^{\prime}t}} \\ & \quad + \mathop \sum \limits_{{\begin{array}{*{20}c} {s^{\prime} \in S} \\ {t - g_{{s^{\prime}s}} > 0} \\ {g_{{s^{\prime}s}} > 0} \\ \end{array} }} Z_{{s^{\prime}st - g_{s^{\prime}s} }} - R_{st} \quad t \in \left\{ { \propto + 1, \ldots ,T} \right\},s \in S, \\ \end{aligned} $$

9 $$ \begin{aligned} X_{st} & = X_{st - 1 } + A_{st} + M_{st} - \mathop \sum \limits_{{\begin{array}{*{20}c} {s^{\prime} \in S} \\ {} \\ {g_{{ss^{\prime}}} \ge 1} \\ \end{array} }} Z_{{ss^{\prime}t}} \\ & \quad + \mathop \sum \limits_{{\begin{array}{*{20}c} {s^{\prime} \in S} \\ {t - g_{{s^{\prime}s}} > 0} \\ {g_{{s^{\prime}s}} \ge 1} \\ \end{array} }} Z_{{s^{\prime}st - g_{{s^{\prime}s}} }} - R_{st} \quad t \in \left\{ {2, \ldots , \propto } \right\},s \in S, \\ \end{aligned} $$

10 $$ \mathop \sum \limits_{s \in F} M_{st} = K_{t} \quad t \in T, $$

11 $$ \mathop \sum \limits_{s \in C} M_{st} = L_{t} \quad t \in T, $$

12 $$ \mathop \sum \limits_{s^{\prime} \in S} Z_{{ss^{\prime } t}} \le Q\quad t \in T,s \in S, $$

13 $$ X_{st} \ge 0;\; Y_{st} \ge 0;\; Z_{{ss^{\prime } t}} \ge 0;\; E_{st} \ge 0;\; B_{st} \ge 0;\; M_{st} \ge 0\quad t \in T,s \in S,s^{\prime} \in S. $$

The aim of the mathematical model (Eq. (1)) is to minimize the total number of used pilots, newly employed pilots, employed captains instead of first officers (it is used for avoiding new employed pilots), transitions and annual leaves with penalizing coefficients $\beta_{s}^{1} ,\beta_{s}^{2} ,\beta_{s}^{3} ,\beta_{{ss^{\prime } }}^{4} \;\;{\text{and}}\;\;\beta_{s}^{5}$, respectively. These coefficients ensure to weight the terms regarding long-term human resources strategies of the firm. Equations (2) and (3) ensure the demand constraints which is calculated by the number of available pilots subtracting by number of annual leaves while considering the number of employed captains instead of first officers. While Eq. (2) provides this balance for first officers, Eq. (3) ensures it for the captains. Equations (4) and (5) provide the holidays considering the number of available pilots at the months for summer and winter months well balanced. The Eq. (4) is the number of holidays given in winter, while Eq. (5) is for summer months. Each equation is multiplied by 0.5, due to the fact that half of pilots should take holidays at winter and summer. Equation (6) is also used by annual leaves; it ensures a ratio of the minimum number of pilots which are using annual leaves in each month for each skill type. Equations (7)–(9) are used for balancing the number of pilots in each month by considering the number of transitions, number of new employment, number of pilots at the previous months and number of retired pilots for different periods. Equations (10) and (11) are used for determining the new employed pilots who are currently under training with non-skill. In practice, if the planning problem has been solved for previous periods, some first officers and captains continue their training. In this case-specific constraints, it is ensured that the first officers and captains, whose training will be completed during the current planning horizon and who have already started training, are assigned to the skills that will be formed according to the aircrafts. Finally, Eq. (12) forces the limitations for a number of transitions in each month considering the capacity of the training academy and Eq. (13) are the valid ranges of decision variables.

### Solution procedure

**Fig. 3:**
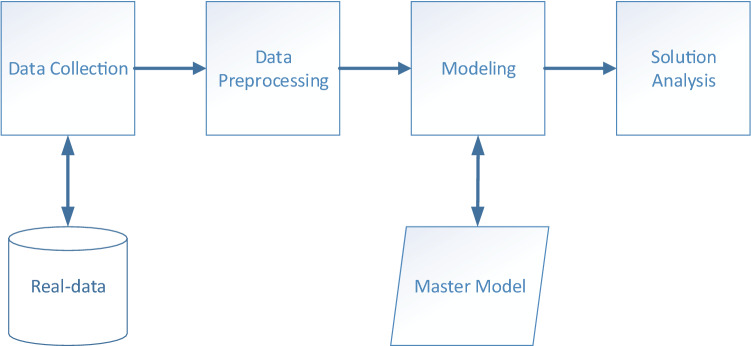
Solution procedure

The solution procedure of the proposed problem is depicted in Fig. 3. First, it is ensured that data are obtained from the real system. Using these data, the calculations and required conversions (e.g., resignation rate) are performed. The master model given in “Master model and notation” section is adapted by entering the data that will constitute input for the modeling. Since the master model is a mixed integer-programming model, the solution of the mathematical model in computer environment is provided with appropriate solvers such as CPLEX. In the last step, the necessary analysis is performed by creating a solution report.

## Experimental results

### Case study

The case study of this study is conducted on an airline company in Turkey. The airline company operates flights to more than 100 destination points. The company serves over 30 million passengers annually on flights to destinations. There are captains and first officers who will work with four different aircraft types to meet this size's passenger demand.

In this airline, there are eight different skills: captains (C) and first officers (F/O) roles of four different plane types. The airline has four different planes, specified by the narrow-body and wide-body planes with two different aircraft. Therefore, the proposed workforce planning system considers different skills and eight skills as a skills set (*S*) from the case study.

**Fig. 4:**
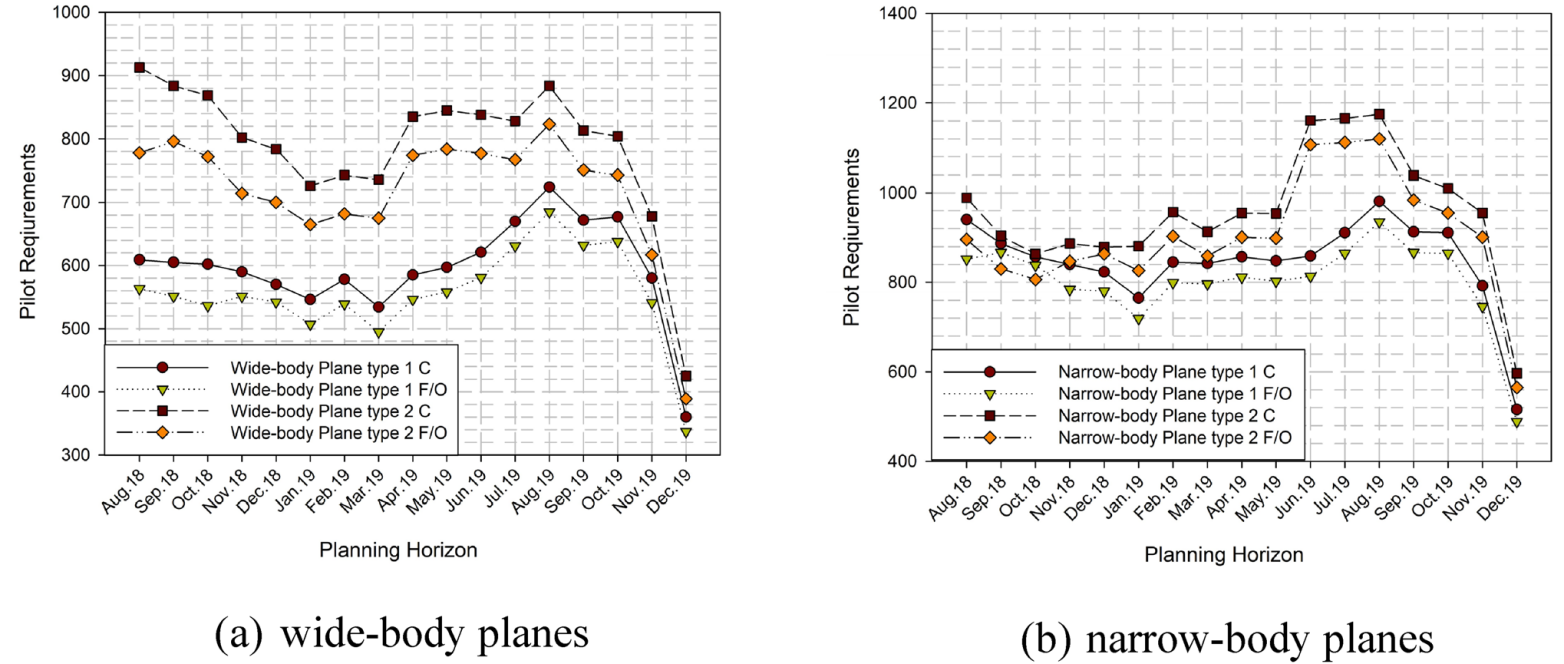
Pilot requirements for each skill type during the planning horizon

Pilot requirements are calculated according to the number of flights handled by each aircraft type. The data obtained from the case study include the flights between August 2018 and December 2019. Pilot requirements (*d*_*st*_) of each skill type during the year are depicted in Fig. 4. It is shown that there appears to be a rapid decline in pilot requirements between November 19 and December 19, as between November 18 and December 18. The reason for this is the decrease in demand during the winter months and the winter season. Therefore, the model tends to make training or holidays to meet the decline in requirements.

The pilot requirements are increasing during the summer months due to Turkey’s tourism condition, and religion tourism’s in the summer months for the wide-body planes. Narrow-body planes’ pilot requirements seem to be increased by a trend, especially for the type 2 planes. The annual leave balancing ratio ($\gamma$) equals 0.1 to balance leaves in each month.

The currently available number of pilots ($h_{s}$) at the starting of the planning period is summarized in Table 2. This table shows that there is are different number of captains and first officers regarding skill types.

**Table 2:** Number of pilots at the starting periods

*h*_s_	Wide-body Plane 1 C	Wide-body Plane 1 F/O	Wide-body Plane 2 C	Wide-body Plane 2 F/O	Narrow-body Plane 1 C	Narrow-body Plane 1 F/O	Narrow-body Plane 2 C	Narrow-body Plane 2 F/O
Starting month	609	563	913	778	940	852	989	896

Duration of training ($\alpha$) for a newly employed pilot equals nine months. Besides, Table 3 presents us the training length between the transitions (*X* means the transition cannot be done). The training facility has a finite capacity (*Q*) for each month’s transitions which equals sixty pilots.

**Table 3:** Training length of the transitions ($g_{{ss^{\prime } }}$)

$g_{{ss^{\prime } }}$	Wide-body Plane 1 C	Wide-body Plane 1 F/O	Wide-body Plane 2 C	Wide-body Plane 2 F/O	Narrow-body Plane 1	Narrow-body Plane 1	Narrow-body Plane 2	Narrow-body Plane 2
					C	F/O	C	F/O
Wide-body Plane 1 C	*X*	*X*	*X*	*X*	*X*	*X*	*X*	*X*
Wide-body Plane 1 F/O	*X*	*X*	*X*	*X*	3	*X*	3	*X*
Wide-body Plane 2 C	*X*	*X*	*X*	*X*	*X*	*X*	*X*	*X*
Wide-body Plane 2 F/O	*X*	*X*	*X*	*X*	2	*X*	3	*X*
Narrow-body Plane 1 C	3	*X*	2	*X*	*X*	*X*	*X*	*X*
Narrow-body Plane 1 F/O	*X*	3	*X*	2	2	*X*	*X*	*X*
Narrow-body Plane 2 C	3	*X*	3	*X*	*X*	*X*	*X*	*X*
Narrow-body Plane 2 F/O	*X*	3	*X*	3	*X*	*X*	2	*X*

Finally, the given data emphasize to the planning pilot workforce is essential for the company during the periods to avoid an excess and insufficient number of pilots.

## Results

**Fig. 5:**
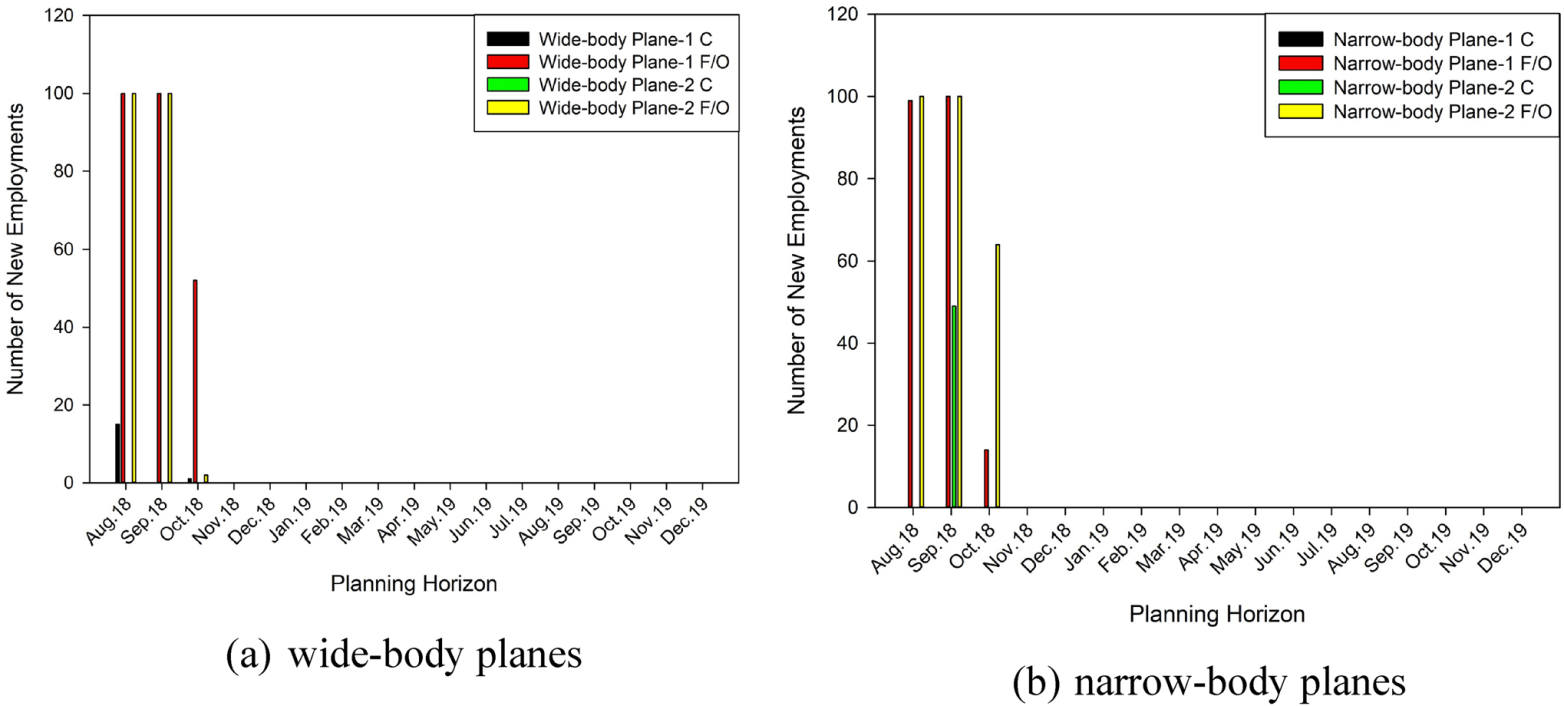
Number of newly employed pilots during the planning horizon

To solve the proposed model, GAMS Optimization software with CPLEX mixed integer programming solver is used for building the mathematical model using a computer that has Intel i7 3.2 GHz and 16 GB RAM. All computations are evaluated less than 3 min. The number of newly employed pilots during the planning horizon is shown in Fig. 5 and given in Table 4.

**Table 4:** Number of newly employed pilots during the planning horizon

*E*_st_	Aug. 18	Sep. 18	Oct. 18
Wide-body Plane 1 C	15	0	1
Wide-body Plane 1 F/O	100	100	52
Wide-body Plane 2 C	0	0	0
Wide-body Plane 2 F/O	100	100	2
Narrow-body Plane 1 C	0	0	0
Narrow-body Plane 1 F/O	99	100	14
Narrow-body Plane 2 C	0	49	0
Narrow-body Plane 2 F/O	100	100	64

It can be understood from Fig. 5 that first officers who have a skill of narrow-body plane are most hired pilot type due to the airline policy. It means that most of the captains are trained from the current first officers. In addition to that, it seems that captains are hired when a transition from other skill types cannot be possible due to the pilot requirements and current hired number of pilots for each skill.

**Fig. 6:**
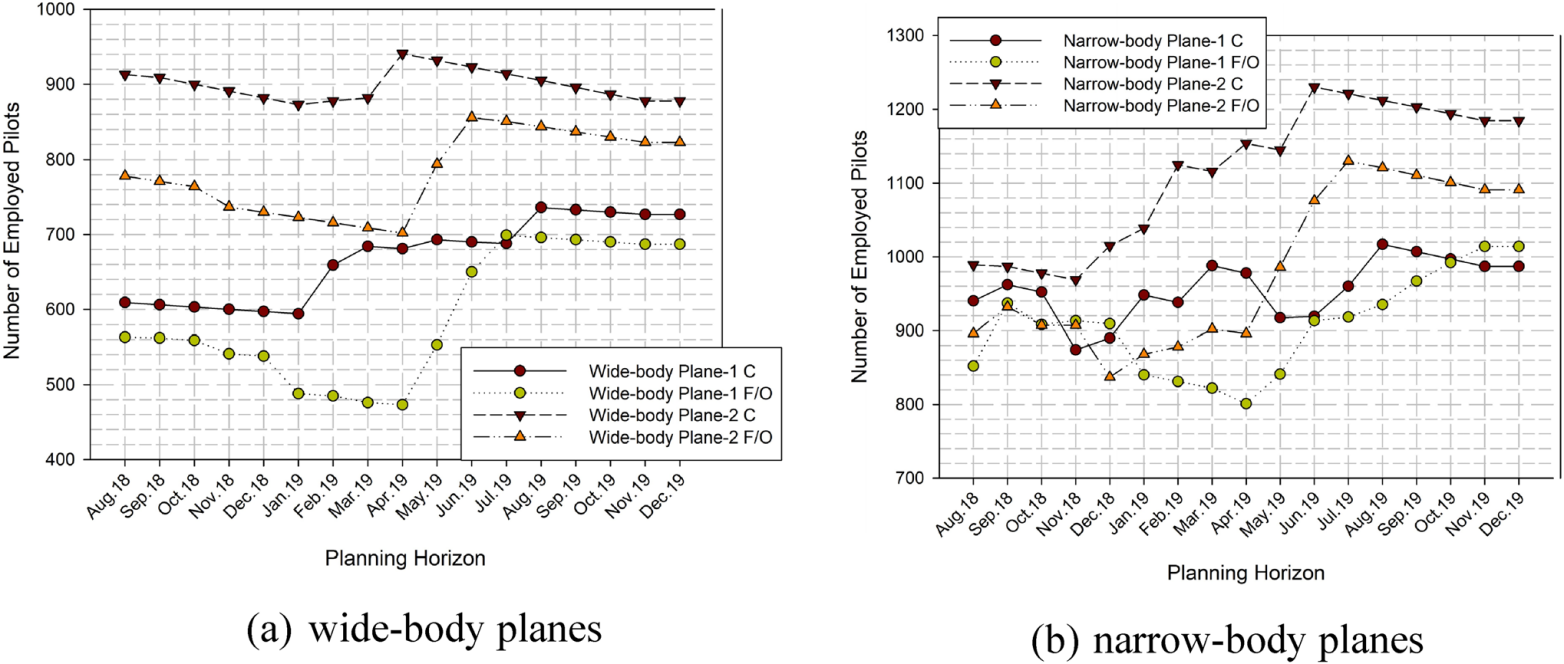
Number of pilots for each month

The employed pilots for each month is shown in Fig. 6. This figure is generally overlapped with the pilot requirements as expected. However, it is a slice difference between requirements and employment on the narrow-body first officers. It is also reasonable due to two rules as follows: (1) captains can be employed temporarily instead of F/Os, and (2) pilots are often trained as a first officer and it may generate a pilot surplus on the first officers.

Table 5 summarizes the results of the mathematical model. According to this table, some observations can be done as follows:

- When the deviation of the pilot requirements is increased during the planning horizon, the model generally reacts with slice deviations. It is provided by the different types of employment options, transitions and temporarily captain-first officer changes.
- The model tries to employ fewer captains due to the airline policy, new employment is generally aided by first officers.
- Transitions are fewer when comparing the new employment. It is also understandable that the model wants to balance the deviations on pilot requirements with new employment instead of transitions.
- Transitions are generally made from the first officers. It is also understandable considering the surplus on the first officer employment.

**Table 5:** Results of the mathematical model

Numbers of monthly transition	Standard deviation of pilot requirements	Standard deviation of number of employed pilots	Average number of new employments	Average number of transitions
Wide-body Plane 1 C	78.48	54.98	1	9
Wide-body Plane 1 F/O	75.32	87.75	15	0
Wide-body Plane 2 C	112.83	20.59	0	6
Wide-body Plane 2 F/O	101.07	54.58	12	0
Narrow-body Plane 1 C	100.33	40.40	0	23
Narrow-body Plane 1 F/O	95.83	66.94	13	0
Narrow-body Plane 2 C	137.64	96.27	3	17
Narrow-body Plane 2 F/O	133.04	107.34	16	0

**Fig. 7:**
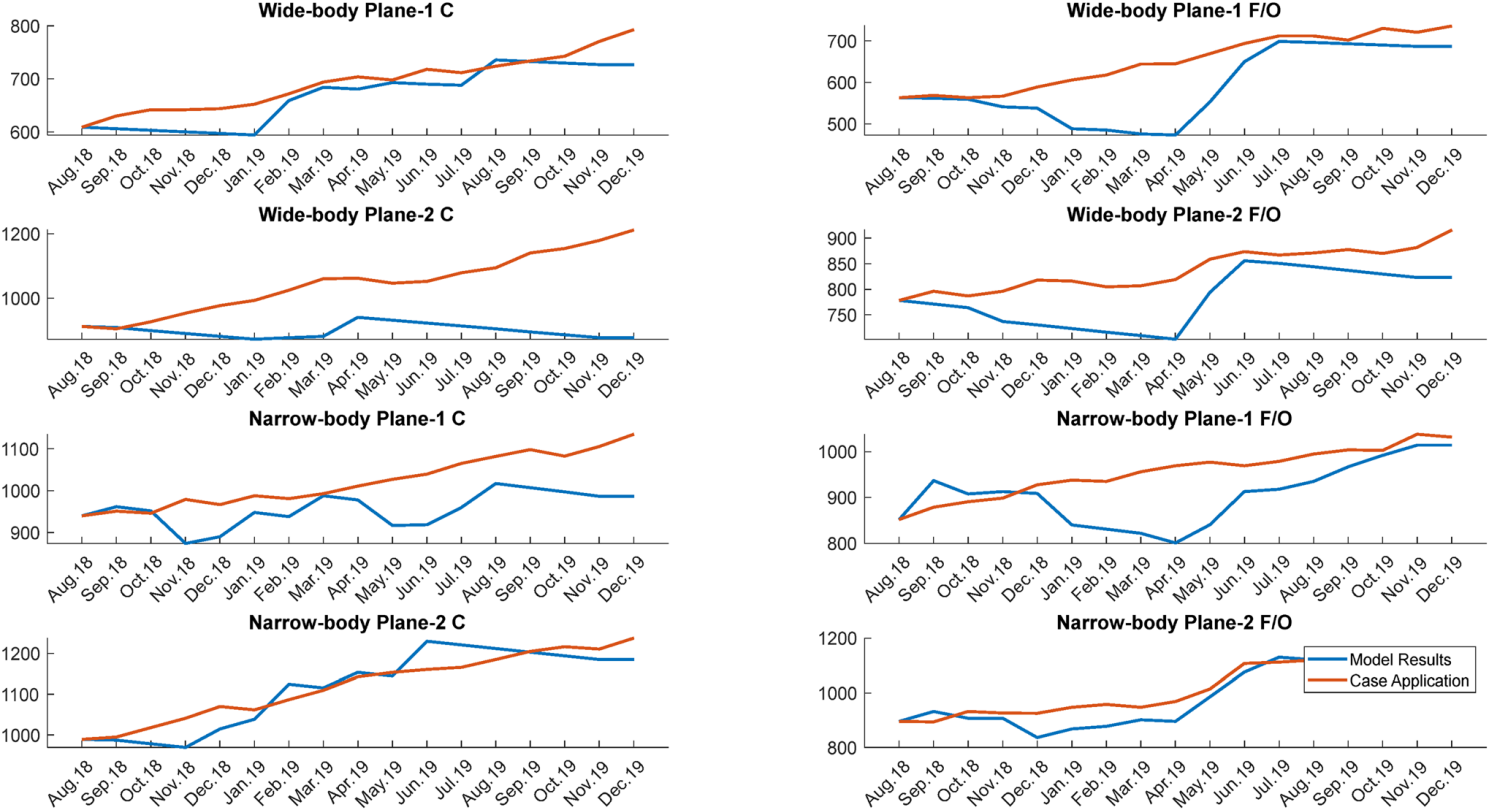
Comparison between case application and model results

When the proposed workforce planning model and the case applications are compared, the number of employed pilots according to their skill types is shown in Fig. 7. It is seen that the proposed model has found quite different results, especially for some pilot skills (i.e., wide-body plane 1 F/O and wide-body plane 2 C). The reason for this is seen as the lower number of employees achieved through transitions. Considering the average values in all skill types in all months, the proposed model improves the system by decreasing the six percent. The improvement value was obtained by dividing the total employment numbers in the case application by the difference between the proposed model results. The most significant improvement was 13% for the wide-body plane 2 C skill, while the lowest improvement was 1% for the narrow-body plane 2 C skill.

**Fig. 8:**
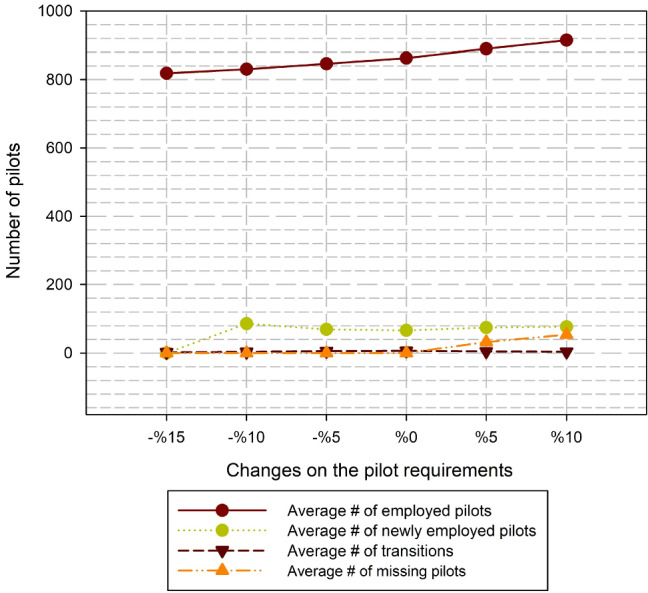
Effects of the changes on the pilot requirements

Finally, it should be analyzed when the pilot requirements are changed how the model fit the new requirement conditions. Figure 8 shows us that the effects of changes on pilot requirements (also a number of available pilots at the starting month). It can be concluded from the figure that the number of employed pilots are increased (or decrease) simultaneously regarding pilot requirements; however, newly employments and transitions are slightly increased due to the ascending on number of available pilots at the starting month. The figure shows that the model is sensitive to differences in the pilot requirements. When the pilot requirements are increased, the model cannot plan the workforce due to the infeasibility on the parameters.

The training length of a new employed pilot may also affect the model results. Basically, when the training time is increased model may decide to decrease transitions and increase new employment. The results are summarized in Table 6. It seems that decreasing training length causes to increase an average number of employment. However, it does not affect the average number of transitions. Furthermore, the new employment decisions are sensitive on the changing at the training length of new employment.

**Table 6:** Effects of the training length

Training length (months)	Average # of employed pilots	Average # of newly employed pilots	Average # of transitions
9	863	66	7
8	867	76	7
7	872	76	7
6	875	76	7
5	880	71	7
4	880	58	7
3	881	71	7

## Conclusion

Workforce planning in the airline companies is highly important to execute all the flights with efficiency. It is hard to determine the number of pilots for an airline company due to some reasons. First, airline companies have different aircraft types, and each aircraft type needs its own skill, it makes this problem complicated. Second, a pilot can be trained for possessing another skill type. Finally, pilot requirements change quietly during the planning horizon. In this paper, a workforce planning problem for an airline company is investigated. The mathematical model considers most of the real-world conditions such as transitions between different skill types considering possible transitions with capacity limitations, determining annual leaves with balancing summer and winter months as the airline policy, considering a minimum number of new employment. The results show us that firm can use transitions instead of new employment and this is a more suitable human resource planning decision. This model can extend different conditions such as new employment limitations, new graduates from different number of flight academies or transitions with different number of options with different costs. A decision support system for such system can be proposed; thereby, the model is solved routinely to manage the entire process. Stochastic parameters can be included to the model such as resignation, demand, etc. Some limitations and challenges of proposed model are given in the following:

- For transitions between types, we can determine who we send to training on an individual basis.
- The need for team planning can be dynamic rather than static, i.e., stochastic modeling.
- We believe that the proposed model solves the problem after one year, whereas we can take the model one-step further and determine the number of pilot employment to run and grow for 3–5 years.

Due to COVID-19 pandemic, future of the aviation is more uncertain. Cargo revenues and profits increase and passenger revenues and profits decrease in this epidemic. This is an opportunity for cargo airlines and for cargo carrying passenger airlines, also a thread for the only passenger carrying airlines. These opportunity and threat make airlines continuously to change their fleet plans. Since pilot transitions are necessary for most of the fleet plan changes, our work becomes more useful for the airlines. There is a huge demand and supply unbalance for the pilots in the aviation industry. There are many new pilots graduated from the flight academics, and many pilots lost their jobs due to pandemic. When the effects of epidemic are over, airlines probably will hire many pilots. This will be a reason for transition of pilots between the fleets. Thus, our work will contribute a lot.

In the future work, we will expand our study to the different field and their applications to the different sectors such as risk analysis, decision-making [41–44].
